# Supplementary material for: Microglia-Based Gene Expression Signature Highly Associated with Prognosis in Low-Grade Glioma
Source: Cancers (Basel). 2022 Sep 30;14(19):4802. doi: 10.3390/cancers14194802 (PMC9564275; doi:10.3390/cancers14194802)
Supplement: Supplementary file 1 [file cancers-14-04802-s001.zip › Supplementary Figures.pdf]

Supplementary Figures

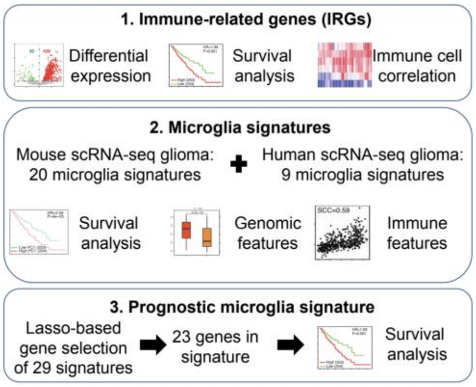

Supplementary Figure S1. Overview of our study.

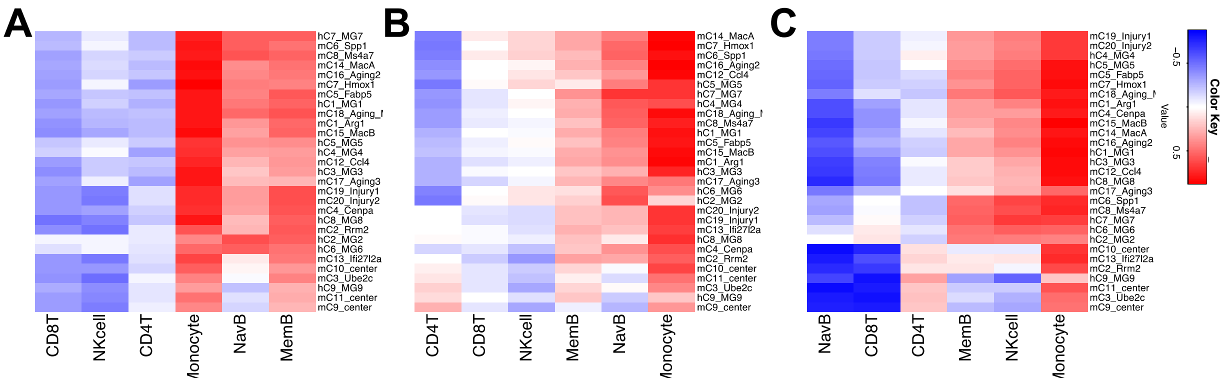

Supplementary Figure S2. Correlation between immune cell infiltration and microglia abundance. **A.** Spearman correlation coefficient in **A.** TCGA LGG, **B.** Rembrandt, and **C.** CGGA.

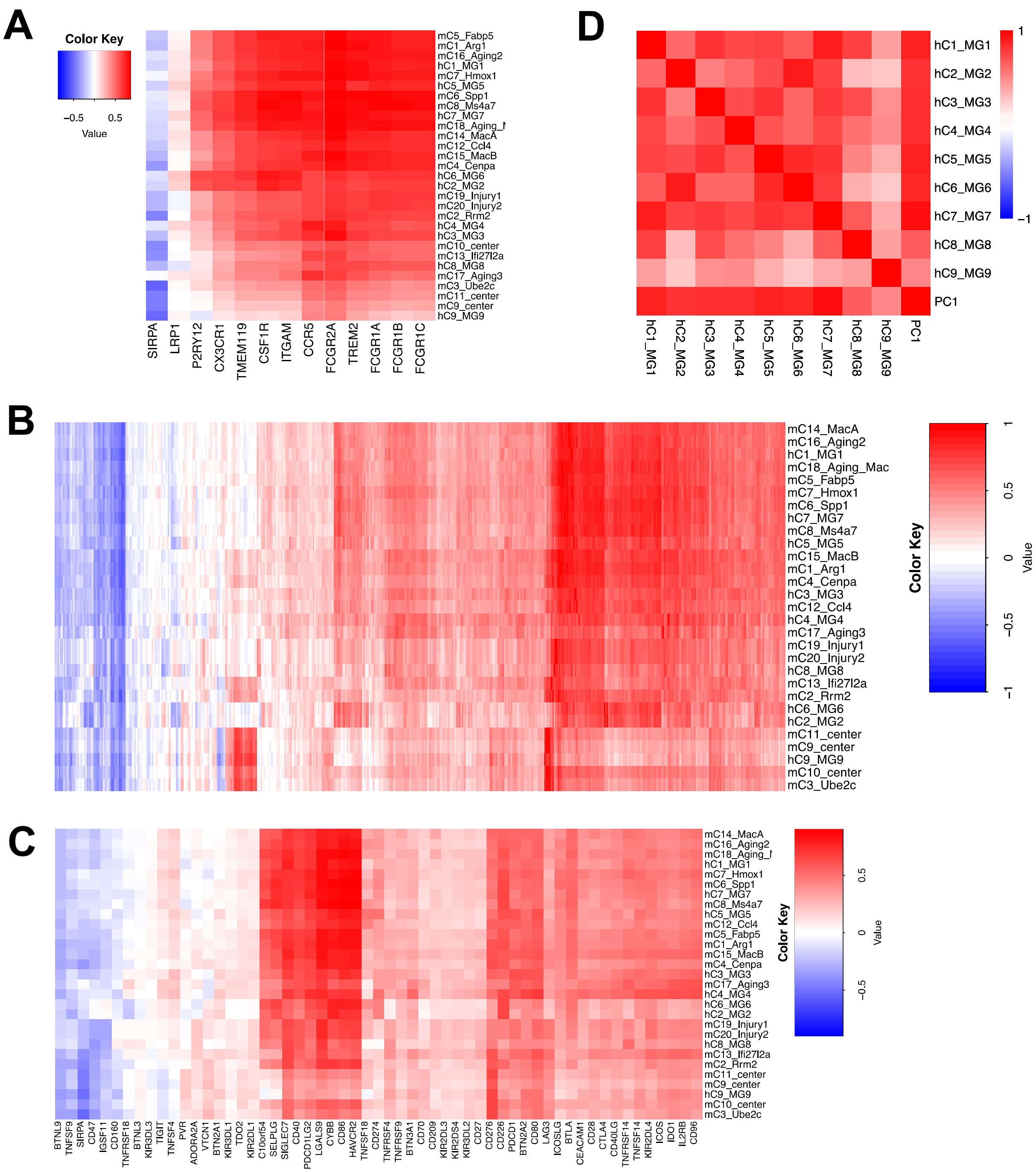

Supplementary Figure S3. Correlation of IRGs with microglia infiltration. A. Spearman correlation coefficient between genes expressed in microglia and microglia abundance. **B.** Spearman correlation coefficient between all significant IRGs and microglia abundance. **C.** Spearman correlation coefficient between all significant immune checkpoint genes and microglia abundance. **D.** Spearman correlation coefficient between PC1 and 9 human microglia signatures.

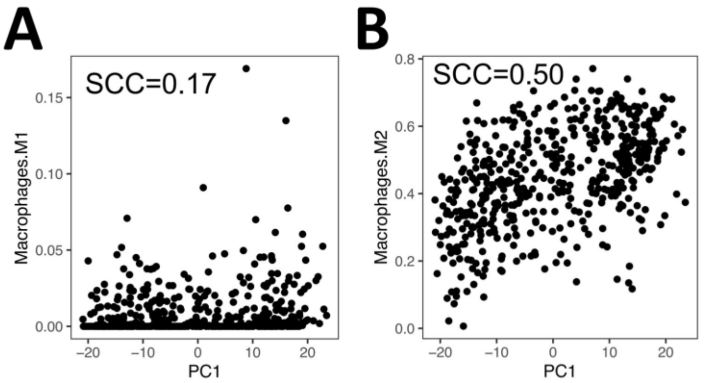

Supplementary Figure S4. Spearman correlation between PC1 and (A) M1 macrophage and (B) M2 macrophage scores.

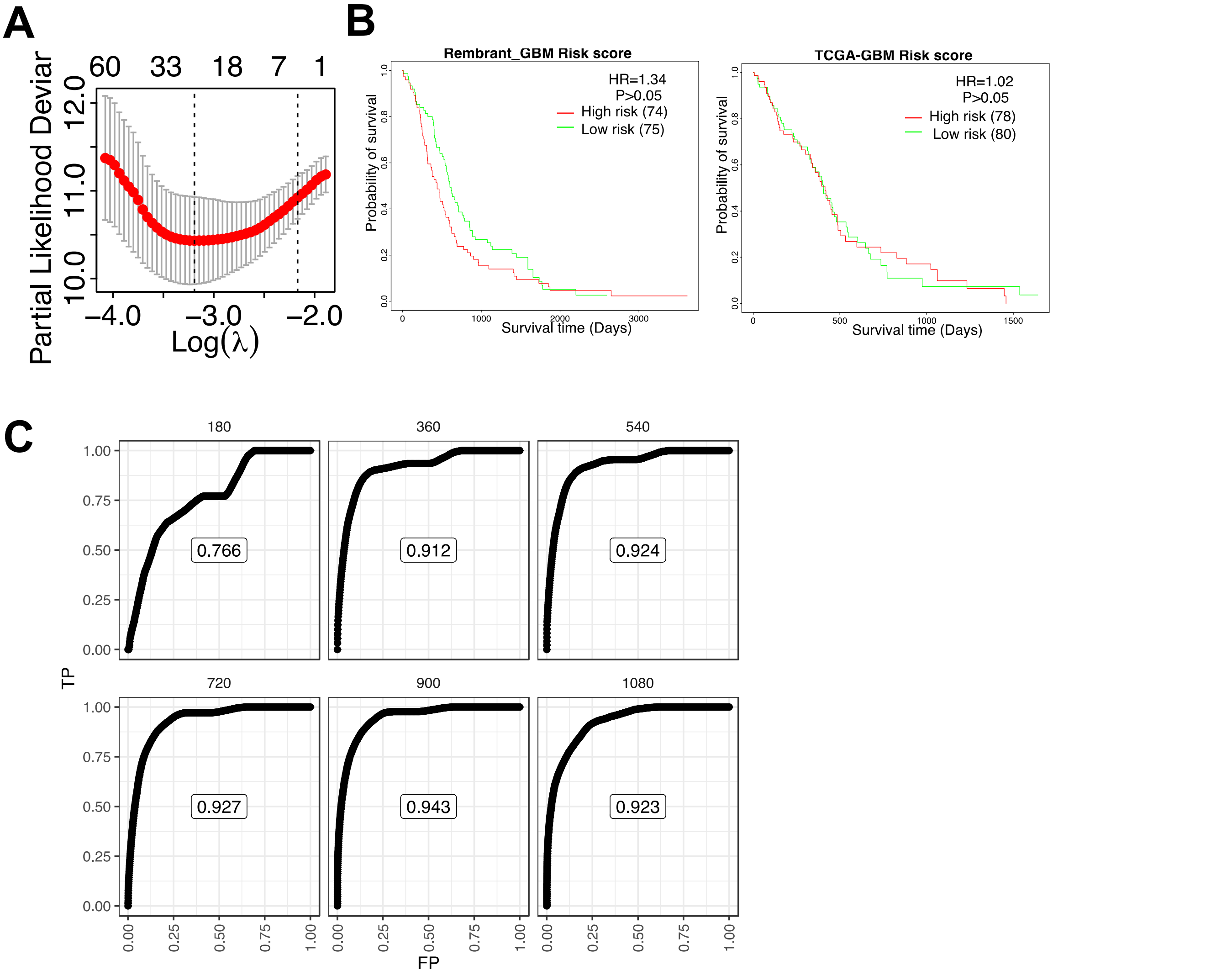

Supplemental Figure S5. Development of a microglia risk score for glioma by LASSO-cox regression analysis. A. Partial likelihood deviance of each lambda value for the LASSO-cox regression analysis. **B.** Kaplan-Meier survival curves between high and low risk patients in GBM in the TCGA and Rembrandt datasets. **C.** Time-dependent ROC of risk score in the TCGA-LGG dataset.
